# The interactions between PML nuclear bodies and small and medium size DNA viruses

**DOI:** 10.1186/s12985-023-02049-4

**Published:** 2023-05-01

**Authors:** Boris Ryabchenko, Vojtěch Šroller, Lenka Horníková, Alexey Lovtsov, Jitka Forstová, Sandra Huérfano

**Affiliations:** Department of Genetics and Microbiology, Faculty of Science, BIOCEV, Charles University, Vestec, 25250 Czech Republic

**Keywords:** PML nuclear bodies, PML, Daxx, Sp100, Sp110, SUMOylation, DNA viruses

## Abstract

Promyelocytic leukemia nuclear bodies (PM NBs), often referred to as membraneless organelles, are dynamic macromolecular protein complexes composed of a PML protein core and other transient or permanent components. PML NBs have been shown to play a role in a wide variety of cellular processes. This review describes in detail the diverse and complex interactions between small and medium size DNA viruses and PML NBs that have been described to date. The PML NB components that interact with small and medium size DNA viruses include PML protein isoforms, ATRX/Daxx, Sp100, Sp110, HP1, and p53, among others. Interaction between viruses and components of these NBs can result in different outcomes, such as influencing viral genome expression and/or replication or impacting IFN-mediated or apoptotic cell responses to viral infection. We discuss how PML NB components abrogate the ability of adenoviruses or Hepatitis B virus to transcribe and/or replicate their genomes and how papillomaviruses use PML NBs and their components to promote their propagation. Interactions between polyomaviruses and PML NBs that are poorly understood but nevertheless suggest that the NBs can serve as scaffolds for viral replication or assembly are also presented. Furthermore, complex interactions between the HBx protein of hepadnaviruses and several PML NBs-associated proteins are also described. Finally, current but scarce information regarding the interactions of VP3/apoptin of the avian anellovirus with PML NBs is provided. Despite the considerable number of studies that have investigated the functions of the PML NBs in the context of viral infection, gaps in our understanding of the fine interactions between viruses and the very dynamic PML NBs remain. The complexity of the bodies is undoubtedly a great challenge that needs to be further addressed.

## Introduction

For small and medium size DNA viruses, the nuclear phase of infection is inevitable given that they do not express all the factors necessary for viral transcription and replication. The nucleus is the densest cell compartment in terms of content. When entering the nucleus, viral genomes must initiate productive transcription and replication while surrounded by heterochromatic regions of the host cell genome as well as several membraneless protein nuclear bodies. Many types of nuclear bodies are known, each with its own morphology and function. These bodies divide the nucleus into distinct environments that facilitate the progression of various biological processes, including the control of gene expression, the processing of RNA transcripts, or the maintenance of DNA replication and repair, among others. The best-characterized nuclear bodies include the nucleolus, polycomb bodies, Cajal bodies, and clastosomes, summarized in [1, 2].

In this review we focus on describing the findings relating to the interaction of small and medium size DNA viruses with promyelocytic leukemia nuclear bodies (PML NBs). Unlike large DNA viruses, small and medium size DNA viruses have limited coding capacity and rely on host nuclear proteins to replicate and transcribe their genomes.

## The structure, composition and function of PML NBs

### PML NBs overview

**Fig. 1:**
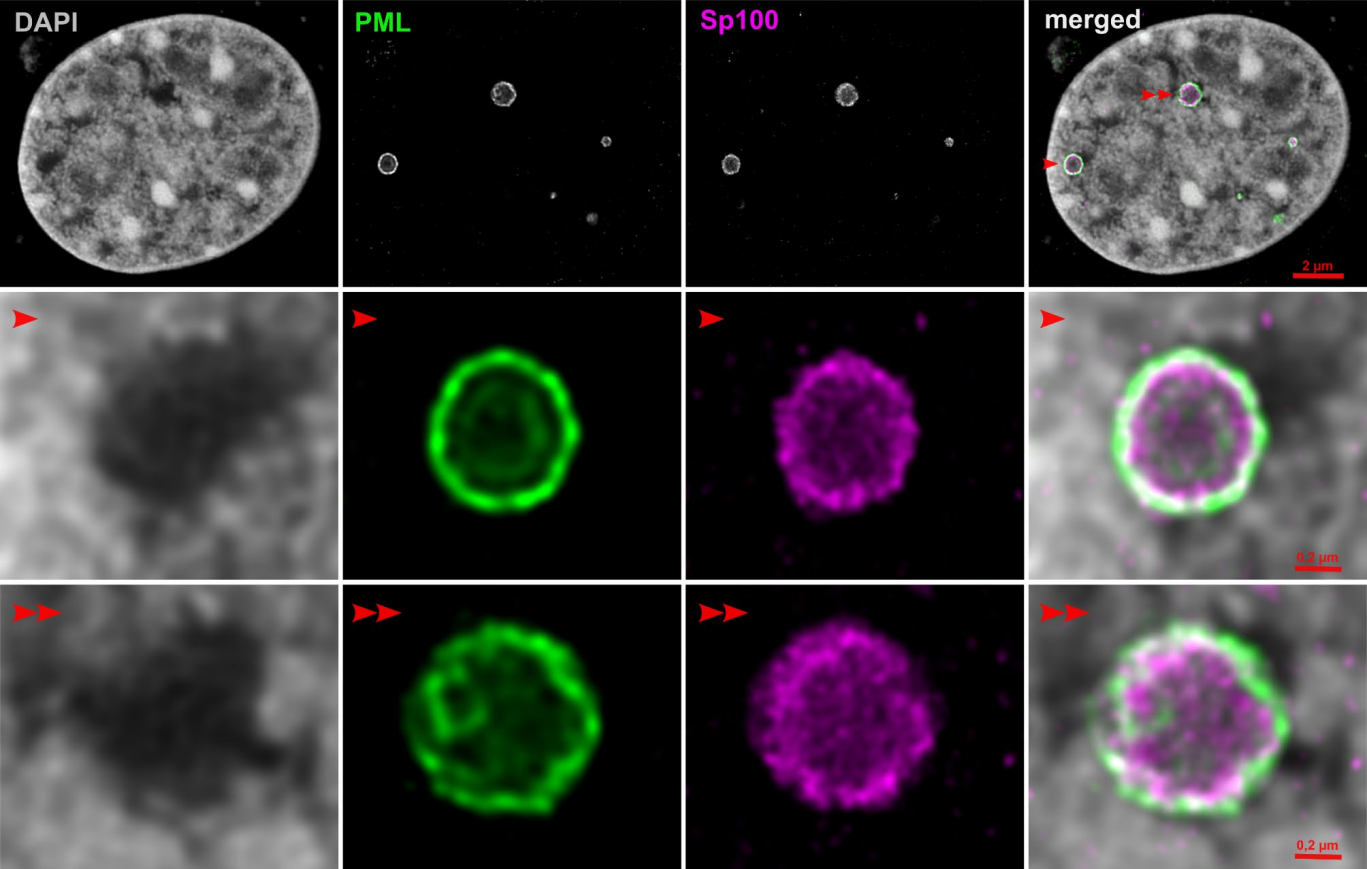
Visualization of PML NBs in the nucleus of a 3T6 cell by STED microscopy

PML NBs, also known as PML oncogenic domain, nuclear domain 10 and Kremer bodies, are distinct, dynamic, nuclear matrix-associated protein complexes. Although they were originally discovered in the early 1960s [3], it was only in the 1990s that the bodies became the focus of great research interest when they were characterized [4] and were found to be associated with the development of acute promyelocytic leukemia [5–7] as well as with sites of genome deposition of several DNA viruses [8]. On electron micrographs, PML NBs appear as spherical, dense structures that are either empty or granular and vary in size from 0.1 to 1 μm [9, 10]. PML NBs are present in the nuclei of almost all types of eukaryotic cells. The numbers and morphology of these structures are dependent on the cell type and the cell cycle phase, and also vary according to the stimulus encountered, summarized in [11, 12]. PML NBs are composed not only of the PML protein but also of other partner proteins. The detailed morphology of the PML NBs and their distribution and localization in the cell nucleus are illustrated in the super-resolution stimulated emission depletion (STED) microscopic image in Fig. 1.

Section through the center of the nucleus of a mouse fibroblast (3T6) cell line showing localization of nuclear chromatin and two constitutive members of PML NB, PML and SP100 proteins (upper panel). Nuclear chromatin is visualized by DAPI staining (confocal microscopy), while PML protein and Sp100 protein (which was transiently expressed as Sp100-EGFP fusion protein) are visualized by indirect immunofluorescence (STED nanoscopy). Cells were stained with I: mouse anti-PML (Enzo, ALX-804-816) and rabbit anti-GFP (Exbio, 11-476-C100) antibodies; followed by II: goat anti-mouse STAR 580 (Abberior, ST580-1001) and goat anti-rabbit STAR 635P (Abberior, ST635P-1002) antibodies. The combined image (merged) is an overlay of channels (DAPI is shown in grey, PML in green, and Sp100-EGFP in magenta). Areas indicated by red arrows are enlarged and presented in the middle and bottom panels. Data were acquired using an Abberior Expert Line STED system (Abberior Instruments) equipped with a Nikon Eclipse Ti-E microscope body and a Nikon CFI Plan Apo Lambda 60× Oil, NA 1.40 objective. All images were deconvoluted using Huygens software (Scientific Volume Imaging B.V.).

The main organizing component of PML NBs is the PML protein that forms scaffolds for binding of the interacting proteins. To date, 271 proteins have been identified as interacting partners of PML NBs [13–15]. While most of them accumulate only transiently at PML NBs under certain conditions, some, such as Sp100, Daxx and SUMO are known to be the most common constitutive residents of these structures [16, 17]. The main PML NB-interacting proteins that have been described in context of small and medium size DNA virus infection are listed in Table 1 and are discussed in the following sections.

**Table 1:** Selected PML NB protein partners and their functions

Name	Main function (reviewed in)	Discovered as PML NBs partner in:
Sp100, Sp110	Recognition of histone post-translational modifications, regulation of gene expression [18, 19]	[7, 20–22]
Daxx	Apoptosis, regulation of transcription, H3.3 chaperone [23, 24]	[25, 26]
ATRX	Chromatin remodeling, H3.3 chaperone [27]	[28]
H3.3	Regulation of transcription [17]	[29]
MRE11 complex	Maintenance of genome stability, response to DNA damage [30]	[31, 32]
p53	Cell cycle regulation, tumor growth suppression, pro-apoptotic transcription factor [33]	[34–37]
HP1	Heterochromatin formation [38]	[39]
SMC5/6	Maintenance of chromatin structure, DNA repair [40]	[41]
HDAC1	Histone deacetylase [42]	[43]
S100A10 (P11)	Maintenance of calcium homeostasis and transduction pathway signaling [44]	[45]
ZNF198	Chromatin remodeling [46]	[47]
SUZ12	Chromatin remodeling [48]	[49]
FOXO4	Transcription factor [50]	[51]

### PML protein

The PML protein is the main organizing molecule of PML NBs. In human cells, the alternative splicing of the primary *PML* transcript, which contains 9 exons, results in theproduction of several PML isoforms. Jensen and colleagues categorized these isoforms into 7 groups, PML I–VII [52]. Although these PML isoforms have an identical N-terminal structure, they differ significantly in the composition of their C-terminus. The PML protein belongs to the family of tripartite motif (TRIM, also known as RBCC)-containing proteins characterized by the presence of three conserved N-terminal domains, namely, a RING finger, a B-box zinc finger (PML possess two, B-box 1 and 2), and a coiled-coil domain [53–58].

The PML protein is characterized by its ability to be covalently modified by the small ubiquitin-like modifier 1 (SUMO1) and SUMO2/3 proteins. Specifically, PML is SUMOylated at Lys65 (localized in the RING finger domain), Lys160 (localized in the B1 box), and Lys490 (present in the nuclear localization signal) [59–62].

SUMO conjugation involves SUMO activating enzyme E1, SUMO conjugating enzyme UBC9 (ubiquitin-conjugating enzyme 9), and SUMO E3 ligase, summarized in [63]. The N-terminal RING finger domain of PML is essential for its interaction with the UBC9 protein [64]. It has also been shown that PML has intrinsic SUMO E3 ligase activity and can thus catalyze its own SUMOylation as well as that of other proteins [65–67]. A wide range of SUMO E3 ligases have been shown to modulate PML activities, among them RanBP2, PIAS1 and ZNF451, reviewed in [68].

In addition, the PML protein contains a SUMO-binding motif (SIM), which is located at the C-terminal part of PML and is independent of the SUMOylation sites [65].

E3 ubiquitin ligases have been found to modify the PML protein and promote its degradation. E6AP, SIAH1 and SIAH2, RNF4, RNF111, UHRF1 and WD repeat 4–containing cullin-RING ubiquitin ligase 4 (CRL4^WDR4^) are examples of E-3 ligases shown to ubiquitinylate the PML protein, reviewed in [62, 68].

Finally, PML is also a phosphoprotein that can be post-translationally modified by serine/threonine kinases [69]. All PML isoforms, except cytoplasmic isoform VII, contain a nuclear localization signal (NLS) and are targeted to the nucleus. Interestingly, isoform I additionally has a nuclear export signal (NES) and can shuttle between the nucleus and the cytoplasm [70].

The amount of PML protein is regulated at the level of transcription and translation in response to various stimuli. For example, type I/II interferons (IFNs) enhance the expression of the PML gene, thereby increasing the number and size of PML NBs. Other regulators of PML include the p53 tumor suppressor protein, p73 (the structural homolog of p53) and pro-inflammatory cytokines, summarized in [12, 71, 72]. To illustrate some of the PML regulation processes the proapoptotic autoregulatory feedback loop between p73, YAP (Yes-associated protein, the transcriptional coactivator) and the PML should be mentioned. The interaction between p73 and YAP, results in the recruitment of YAP to PML NBs where it is SUMOylated. SUMOylation confers greater stability to YAP which in turn promotes the transcriptional activity of p73. PML and pro-apoptotic genes are direct transcriptional targets of p73/YAP [73, 74].

### Formation and dynamics of PML NBs

Several stages of PML NB biogenesis have been described. The first one involves the oligomerization/polymerization of the PML protein, a process that depends on the presence of reactive oxygen species (ROS) and cysteine residues in PMLs. ROS induce intermolecular PML crosslinking *via* the formation of disulfide bridges. Non-covalent interactions mediated by the PML RBCC motif are required for the assembly of oxidized PML. In this case, it has been suggested that RING tetramerization may be the first step in PML NB biogenesis, followed by B1 box polymerization. Furthermore, it was shown that the C-terminal regions of specific PML isoforms may also be involved in the oligomerization/polymerization step of PML NBs. The second phase of PML NB formation entails the recruitment of UBC9, leading to the SUMOylation of PML, which is important for subsequent PML-PML interactions through SUMO-SIM interactions. The final phase involves the formation of SUMO-SIM interactions between the PML scaffold and a large number of client proteins [25, 65, 75–78]. The complexity of PML NB composition is considerably increased by the fact that individual nuclear bodies may be composed of different PML isoforms [12, 70]. PML NBs are anchored in the nucleus by tight interaction with surrounding chromatin [79, 80]. The number of PML NBs remains unchanged until the S-phase of the cell cycle when the fission of PML NBs attached to dividing chromatin fibers results in their duplication [81]. PML NBs meet the criteria of liquid–liquid phase separation process-based structures given that their formation is mainly driven by phase-separation mechanisms [82].

### Functions of PML NBs

PML NBs are involved in chromatin remodeling, the regulation of transcription (e.g., of IFN genes) [83–86], DNA damage responses, the suppression of tumor growth, senescence, apoptotic responses, the regulation of cell cycle progression antiviral defenses, reviewed in [17, 87–89] and the regulation of the alternative lengthening of telomeres, reviewed in [90]. Three major hypotheses have been suggested to explain the multifunctionality of PML NBs, as follows: (i) PML NBs are protein depots that accumulate in specific regions of the nucleus; (ii) PML NBs are nuclear platforms for the SUMOylation and other post-translational modifications of partner proteins; and (iii) PML NBs are active sites of chromatin regulation.

Interestingly, the antiviral functions of PML NBs were identified *via* the observation that they rapidly disappear soon after infection by viruses with large DNA genomes [8]. For example, the early regulatory protein ICP0 of Human alphaherpesvirus 1 (also known as herpes simplex virus 1 [HSV-1]) and its homolog ORF61 in Varicella zoster virus possesses E3 ubiquitin ligase activity, which targets PML and Sp100 for proteasomal degradation [91–93]. The disruption of PML NBs by early viral proteins or tegument proteins has also been described for other herpesviruses (reviewed in [94–97]). Meanwhile, several studies have indicated that some PML NB components can be advantageous for herpesvirus infection and the establishment of latent infection. These observations suggest that PML NBs may play both pro-viral and antiviral roles, reviewed in [98].

A recent discovery points to a novel mechanism that links PML proteins to innate immune responses. It was shown that human PML I isoform contains a vestigial exonuclease domain. The domain lacks the catalytic residues for its activity. Nevertheless, it was shown that the presence of the domain promotes the suppression of the endogenous retroelement, long-interspersed element-1 (LINE-1). Suppression of LINE-1, was shown to be dependent on the ability of PML to shuttle to the cytoplasm via NES [99]. Thus, since LINE-1 has been shown to be substrate for the DNA sensor, Cyclic GMP–AMP synthase (cGAS) [100], PML I can negatively regulate cGAS-STING pathway upon conditions when LINE-1retrotransposons are up-regulated.

Next, we discuss current knowledge relating to the interactions of small and medium size DNA viruses with PML NB components and their functional significance. Highlights of the described interplay of individual DNA viruses with PML NBs are presented at the end of each section.

## Small and medium size DNA viruses and their interactions with PML NBs

### Interactions between adenoviruses and PML NBs

The family *Adenoviridae* comprises medium size, non-enveloped viruses with linear, double-stranded DNA (dsDNA) genomes ranging from 25 to 48 kbp in length. Their genomes are packed into an icosahedral capsid that has a diameter of approximately 95 nm and fibers protruding from its vertices. Adenoviruses (AdVs) have been isolated from a variety of vertebrate hosts, reviewed in [101]. Although AdV infections in humans are mostly subclinical, some types are associated with pathologies, such as acute respiratory infections or acute gastroenteritis. After internalization by the cell, AdVs use the endosomal pathway for trafficking to the nucleus, where the viral core is delivered after uncoating, and also where viral transcription, replication, and assembly take place. AdV proteins are denoted as early (E), intermediate (I), or late (L), depending on the phase of infection in which they are produced.

AdV infection results in the remodeling of spherical PML NBs into fibrous structures, a process that requires the viral protein E4-ORF3, which directly interacts with PML isoform II (PML-II) [102–104]. E4-ORF3 can form dimers that assemble into linear and branched oligomer threads *via* the exchange of their C-terminal tails. E4-ORF3 oligomers form a 3D network in the nucleus around viral replication domains, resulting in avidity-driven interactions with PML [105]. Moreover, emergent E4-ORF3 oligomers serve as a binding interface for MRE11/RAD50/NBS1 (MRN) DNA repair complexes. The sequestration of this complex during infection prevents its activity, which leads to the formation of AdV genome concatemers during replication. In addition, it has been shown that AdVs avert genome concatemerization by targeting the MRN complex not only through E4-ORF3 but also *via* the viral proteins E4-ORF6 and E1B-55 K [106, 107].

Several studies have provided insight into the molecular details underlying how MRN is inactivated during infection. Initially, during infection, MRE11 and other cellular proteins, such as p53, are subjected to proteasomal degradation by a multiprotein complex with E3 ubiquitin ligase activity. This complex is formed by the viral proteins E4-ORF6 and E1B-55 K and the host cell proteins elongin B/C, CUL5, and RBX1 [108, 109]. Subsequently, MRN complexes are exported from the nucleus to the cytoplasm and sequestered in aggresomes composed of E1B-55 K [110]. It was proposed that, initially, E4-ORF3 and MRN are localized to the nucleus bound to PML NBs of altered morphology. Following the recruitment of E1B-55 K to the PML NBs, the proteins traffic to the cytoplasm, where E1B-55 K forms aggresomes that sequester MRN, thereby inhibiting its function and accelerating the ubiquitination-dependent proteasomal degradation of MRE11 [110].

It has been reported the MRE11 and Nbs1 are transiently SUMOylated during AdV type 5 infection. This modification was observed following E4-OFR3-mediated MRN translocation and was proposed to facilitate the degradation of the MRN complex [111]. E4-ORF3 can also function as a viral SUMO E3 ligase and E4 elongase [112], while E4-OFR3 complexes can sequester the host cell E3 SUMO ligase PIAS3 [113].

Importantly, Ullman and Hearing showed that in the absence of functional E4-ORF3, an antiviral state induced in the cell by IFN-α or IFN-γ leads to significant inhibition of AdV genome replication, effects that can be reversed by PML protein downregulation [114]. This suggests that the E4-ORF3-mediated disruption of PML NB integrity likely represents a viral defense mechanism against the antiviral activities of the NBs. This function of E4-OFR3 is conserved among AdV serotypes [115].

Schreiner et al. [116] showed that the Daxx protein negatively regulates AdV type 5 replication and that, during infection, Daxx undergoes proteasomal degradation in an E1B-55 K-, but not E4-OFR6-, dependent manner. Later, it was shown that upon infection, E1B-55 K co-localizes with RING finger protein 4 (RNF4), a cellular SUMO-targeted E3 ubiquitin ligase, in specific insoluble aggregates in the nucleus, which mediates the interaction between Daxx and RNF4, leading to Daxx degradation [117]. Also, it has been demonstrated that ATRX can suppress AdV replication. The ATRX/Daxx complex localizes to the promoter regions of the AdV genome during infection. As Daxx does not possess a DNA-binding domain, ATRX likely links the complex to chromatin. The repressive function of Daxx and ATRX is then mediated *via* the recruitment of histone deacetylases (HDACs). In the absence of ATRX/Daxx, reduced genome condensation enables a more efficient expression of AdV genes. Unlike for Daxx alone, but like for the MRN complex, the degradation of the ATRX/Daxx complex is mediated by the E1B-55 K/E4-ORF6 E3 ubiquitin ligase complex [118]. Moreover, adenoviral capsid protein VI also suppresses Daxx activity. A subpopulation of capsid protein VI molecules is targeted to the nucleus, localizes in the vicinity of PML NBs, interacts with Daxx, and may be involved in its translocation to the cytoplasm [119]. Meanwhile, Daxx is responsible for the repression of the immediate early E1A promoter, which functions as the key regulator of viral transcription and replication, reviewed in [120].

AdV transactivating protein E1A-13 S is essential for activating viral transcription in the early phase of infection. During infection, the protein is localized to PML NBs and interacts with PML-II. Such interaction positively regulates the transcription of viral genes. Interestingly, mutation of the SIM motif of PML-II increased its viral gene transactivating activity. This suggested that, as the PML SIM has been shown to mediate noncovalent interactions with other SUMOylated proteins as well as other PML isoforms, the coactivator properties of PML-II do not depend on its localization to PML bodies [121].

During AdV infection, only the Sp100A isoform of Sp100, but not isoforms Sp100B, Sp100C, or Sp100-HMG, is localized to PML NBs of altered morphology. A significant number of remodeled PML NBs containing Sp100A are found in association with the outer rim of viral replication centers (RCs), whereas other Sp100 isoforms accumulate exclusively within RCs. The C-terminal domains of these longer Sp100 isoforms are likely responsible for this differential localization. Newly synthesized viral RNA is also localized to the outer parts of adenoviral RCs. In addition, Sp100A binding to heterochromatin protein 1 alpha (HP1α) is reduced during infection. It has been proposed that the Sp100-HP1α complex functions as a repressor of viral replication *via* a chromatin condensation-based mechanism and that AdV counteracts the repressive function of the complex by disrupting its integrity[121]. In detail, Berscheminski et al. found that Sp100A alone can act positively on adenoviral promoters when located in disrupted PML NBs, possibly by recruiting histone acetylases (HATs) and thus creating a transcriptionally favorable environment. The authors suggested that Sp100A is retained within disrupted PML NBs surrounding RCs, that is, at sites of active viral transcription, whereas Sp100B, Sp100C, and Sp100-HMG are displaced from these regions and cannot suppress transcription. AdVs, therefore, not only counteract mechanisms of innate immunity but also hijack PML NB components. Fewer interactions between Sp100 and HP1α might also result from decreased SUMOylation of Sp100. Current evidence supports a model whereby AdVs induce the deSUMOylation of specific components of PML NBs, which prevents the localization of viral transcriptional repressors, such as HP1, Daxx, and ATRX, to disrupted PML NBs [122].

Adenoviral DNA-binding protein E2A is SUMOylated during infection and likely connects viral RCs with disrupted PML NBs. The post-translational modification of E2A does not affect its ability to bind to the viral genome. E2A binds Sp100A, which, in turn, increases the number of E2A molecules in the vicinity of transcriptionally active sites and positively affects viral gene expression [123]. This association has been observed 4 h post-infection, once the E2A protein has been synthesized [124].

In summary, during AdV infection, PML NBs components are targeted by the virus as they mostly represent a barrier to viral infection. However, Sp100A promotes viral transcription and PML-II can either restrict or promote infection by interacting with different viral proteins.

Box 1. Highlights of the interplay between adenoviruses and PML NBs

1. E4-ORF3 binds to PML-II and induces PML NB remodeling -“disruption”.
2. E4-ORF3, E4-ORF6, and E1B-55K target/remodel PML to (a) promote the degradation of MRE11, thus preventing genome concatemerization; and (b) promote the degradation of ATRX/Daxx, thereby preventing repression of replication.
3. Protein VI interacts with Daxx thereby inhibiting its repressive activity.
4. PML-II binds to the viral transactivator protein E1A-13S, which promotes its activity.
5. SP100A localizes to the outer part of viral replication centres and promotes the transcription of the viral genome.

### Interactions between papillomaviruses and PML NB components

Papillomaviruses (PVs) are small DNA tumor viruses with non-enveloped icosahedral capsids of approximately 52–55 nm in diameter. PVs have circular dsDNA genomes (approximately 8 kbp) packaged as a minichromosome with cellular histones. This group of viruses infects a wide range of vertebrates, from birds to mammals. To date, at least 450 human papillomavirus (HPV) genotypes have been identified [125]. Although infection by some PVs leads to the production of benign warts, other PVs can induce cancer [126, 127]. PVs infect basal keratinocytes *via* skin microlesions, and these cells serve as reservoirs for infection. The infected keratinocytes contain only a few copies of the viral genome, which are present as episomes. The full life cycle of PVs relies on the terminal differentiation of these cells [128, 129]. Owing to the complexity of the PV life cycle, PV propagation in tissue culture is challenging. Consequently, most related studies have employed models simulating different phases of infection, such as the overexpression of individual viral proteins, pseudovirus infection, or PV genome transfection.

PV genomes are composed of E and L coding regions separated by control region sequences. The E region encodes up to eight proteins (E1 to E8), depending on the virus type. E1 and E2 are important for genome replication and transcription, while E6 and E7 are viral oncoproteins. The L region encodes the capsid proteins L1 and L2 [130].The infection of basal epithelial cells by the virus is followed by the initial amplification of the genome. In this process, the PV genome is replicated until an optimal copy number is reached, followed by a maintenance phase in which a constant PV genome copy number is maintained. Later, a massive amplification of the viral genome and virion assembly occurs in terminally differentiated cells [128, 131, 132].

PVs traffic to the nucleus within endocytic vesicles in the endosomal pathway. The virions undergo remodeling in which the L2 protein is arranged in a transmembrane configuration. Until recently, the remodeled subparticle was thought to be composed of L2 protein and viral DNA with histones [133], reviewed in [134]. However, it has since been demonstrated that L1 proteins, likely in capsomer form, are also components of the subviral particle (L2-L1/vDNA complex) [135]. After endosomal sorting, the L2-L1/vDNA complex travels to the *trans*-Golgi network (TGN) by retrograde transport [136, 137]. The nuclear import of the complex depends on mitotic nuclear envelope breakdown [138]. It was recently found that viral subparticles in transport vesicles enter the nucleus and remain “covered” for a short time after the completion of mitosis [135, 139].

The association of viral proteins with PML NB components was observed in studies where viral proteins were overexpressed. First, the minor capsid protein L2 of bovine PV (BPV) was shown to be essential for attracting the major structural protein L1 and the non-structural protein E2 to PML NBs [133]. Subsequently, Florin et al. found that the HPV L2 protein induced PML NB reorganization [140]. Specifically, the authors reported that Sp100 was excluded from the PML NBs, as well as the massive recruitment of Daxx protein, while the spot-like shape of the bodies was preserved. Further studies showed that the L2 protein binds to Daxx *via* its C-terminal region [141, 142]. Finally, it was found that, when overexpressed in human keratinocytes, the E4 protein forms inclusions that are surrounded by PML protein [143].

Next, several experiments using pseudoviruses or even viruses provided clear evidence that interactions between viral proteins and PML NBs are required during the early stages of infection. Using BPV pseudovirions, Day et al. demonstrated that after cell entry, remodeled pseudovirions that were transported to the nucleus co-localized with PML NBs [144]. Furthermore, it was shown that the expression of the gene that was packaged in the pseudovirions was decreased in infected PML knockout cells. The positive role of PML NBs in the life cycle of the virus was confirmed by experiments using BPVs isolated from bovine warts [144]. Additionally, Bienkowska-Haba et al. demonstrated that PML NBs confer a protective environment for the HPV pseudogenome [145]. Specifically, the authors found that the amount of EdU-labelled DNA delivered by HPV16 particles was greatly reduced in PML knockdown HaCaT cells compared with that in the parental cell line.

Details regarding the interaction between PML NB components and PVs have gradually emerged. Bund et al. [146] identified a SIM motif at position 286–289 of the L2 capsid protein that is responsible for the localization of L2 in PML NBs. In agreement with this, when the L2 SIM motif was mutated, neither entry nor sorting of pseudoviruses was affected; in contrast, L2-L1/vDNA complexes did not accumulate in the PML NBs. The authors concluded that L2 interaction with SUMO-2 proteins is required for the localization of the viral L2-L1/vDNA complex with PML NBs. The importance of this motif was later also independently corroborated [147]. Next, Kivipold et al. [148] to understand the role of Daxx in the PV life cycle, transfected U2OS cells with HPV genomes and investigated the localization of the RCs relative to that of PML and Daxx. They found that RCs are localized near PML NBs. Furthermore, Daxx downregulation negatively affected the expression of early genes and, consequently, viral genome replication. Finally, Schweiger et al. [149] recently showed that the autophagy receptor p62 binds to subviral particles in endosomes and accompanies them to the nucleus, where hybrid p62-subviral particle-PML assemblies are detected. The authors suggested that the interaction between subviral particles and p62 exerts a protective effect on the viral genome. p62 depletion resulted in the degradation of L2 and a decrease in the expression of the reporter gene carried by the pseudovirions [149].

Notably, despite the overall beneficial effect of the association between PVs and PML NBs for the virus, it has also been demonstrated that Sp100, a PML NB component, plays a negative role in the early and late phases of the viral life cycle. First, it was shown that the transfection of Sp100-depleted immortalized primary human keratinocytes with the PV genome resulted in enhanced viral transcription and replication and increased the immortalization of keratinocytes [150]. Further studies showed that Sp100 not only associated with the replication foci formed upon differentiation in a HPV-containing cervical cell line (derived from a HPV31-positive cervical biopsy) but also mediated the repression of late HPV31 transcription and reduced viral replication in the differentiated cells. Chromatin immunoprecipitation studies showed that Sp100 binds at multiple sites in the viral genomes, implying that Sp100 binding is sequence-independent [151].

Recently, Guion et al. used high-resolution microscopy in combination with methods for differential membrane permeabilization to understand the dynamics of the interactions between PML NB components and HPV pseudovirions. They followed the co-localization of membrane-associated or naked subviral particles with PML, SUMO-1, and Sp100 at early and late interphase. In early interphase, large cytosolic PML protein aggregates could be seen; however, the pseudovirions only co-localized with PML in the nucleus when PML was recruited back to the nucleus after mitosis, and this association with PML continued throughout interphase. Moreover, the genomes, still enclosed in transport vesicles, co-localized with PML and SUMO-1 in the nucleus, while Sp100 was recruited to the pseudogenomes only after they had shed their transport vesicles, which occurred during late interphase [147].

In conclusion, for PVs, it has been demonstrated that PML NB components mostly promote viral infection *via* several mechanisms. Nevertheless, one PML NB component, Sp100, has been shown to restrict viral transcription and replication in the later stages of infection.

Box 2. Highlights of the interplay between papillomaviruses and PML NBs

1. The viral capsid protein L2 interacts with PML via its SIM motif.
2. PML and SUMO-1 are recruited to transport vesicles containing subviral particles, thus conferring protection to the viral genome in the nucleus.
3. Daxx positively regulates viral gene expression.
4. In the nucleus, once the subviral particles are released from transport vesicles, they interact with Sp100. This interaction negatively influences viral genome transcription and replication.

### Interactions between polyomaviruses and PML NB components

Polyomaviruses (PyVs) are small, non-enveloped icosahedral viruses (~ 45 nm in diameter) with circular dsDNA genomes (~ 5 kbps) packaged as a minichromosome with cellular histones. The viral genomes encode early, so-called tumorigenic T antigens—multifunctional proteins that play a role in the regulation of gene expression as well as in the modulation of the host cell immune response and tumorigenesis—and late gene products, namely, the capsid proteins VP1, VP2, and VP3 [152–154]. Primate PyVs encode an additional late protein, a helper phosphoprotein called agnoprotein, the function of which is incompletely understood [155].

PyVs are widespread in nature. They primarily infect mammals and birds, causing predominantly persistent, asymptomatic infections. The most studied PyVs are model viruses—mouse polyomavirus (MPyV), simian virus 40 (SV40), and human BK (BKPyV) [156], JC (JCPyV) [157], and Merkel cell (MCPyV) [158] polyomaviruses. Immunodeficiency or immunosuppression allows the reactivation of these viruses. When this happens, BKPyV causes an opportunistic infection of the kidneys, which may result in nephropathy. JCPyV can cause the brain disease progressive multifocal leukoencephalopathy, while MCPyV is associated with a rare type of skin cancer called Merkel cell carcinoma.

PyVs enter cells by receptor-mediated endocytosis and travel in endosomes to the endoplasmic reticulum. They are subsequently released into the cytosol, from where they translocate to the nucleus through nucleopores, reviewed in [159]. In the nucleus, they use host cell functions for early and late gene transcription, alternative splicing, and genome replication. However, the role of PML NBs in PyVs propagation remains poorly understood.

In the early stage of SV40 infection, viral DNA and large T antigen (LT) were observed in close proximity to PML NBs [8, 102, 160]. Although individually expressed SV40 LT antigens have been found to co-localize with PML NBs [102, 161], active replication of viral DNA is required for the localization of viral DNA close to PML NBs [160, 162]. Thus, although SV40 replicates its genomic DNA close to PML NBs, no alterations or modifications of the NBs during infection have been detected [8]. Interestingly, similarly to AdV E4-ORF6 and E1B-55 K of adenovirus, the SV40 large T antigen targets the MRN complex and disturbs the formation of nuclear foci containing MRE11 [163, 164]. Whether PML NBs play a role in this process is unknown.

In contrast, infection with other PyVs was shown to result in significant alterations and modifications to PML NBs during infection. BKPyV alters the number and size of PML NBs during infection. In BKPyV-infected cells, there are fewer but larger PML NBs compared with that seen in non-infected cells [165]. Moreover, LT antigen was found to co-localize with PML NB structures, whereas viral DNA was observed juxtaposed to PML NBs [160, 165]. In addition to number and size, BKPyV infection also influences the composition of PML NBs. For instance, PML and SUMO-1 were reported to be associated with PML NBs during the whole course of infection, whereas Sp100 and Daxx dissociated from NBs in the late phase of infection [165]. Although active replication of viral DNA is indispensable for PML NB reorganization and viral DNA association with PML NBs, intact PML NBs are not required for virus replication. PML protein knockdown leads to the complete disruption of PML NB structures without affecting viral titer or viral protein levels [165]. Similar effects have been observed in cells infected with other PyVs. Specifically, PML NBs are larger in MPyV infected cells than in non-infected cells. Furthermore, whereas LT antigen localizes close to PML NBs, MPyV DNA localizes adjacent to and in NBs. As with BKPyV infection, PML NBs are not required for MPyV replication [166]. Enlarged, sphere-shaped PML NBs were also detected in cells transfected with a replication-competent MCPyV genome [167]. In MCPyV-infected cells, LT antigen was detected in proximity to PML NBs, and an increase in the number and size of the NBs was also observed. Moreover, no Sp100 signal was detected in approximately 30% of the NBs evaluated, indicative of PML NB reorganization. Although the knockdown of PML had little effect on the efficiency of viral genome replication, Sp100 knockdown resulted in increased replication of MCPyV genomes, suggesting that Sp100 plays a restrictive role of in virus replication [167].

The most prominent reorganization of PML NBs was seen in JCPyV-infected cells. PML NBs were found to be larger in the late phase of JCPyV infection both in vitro and in oligodendrocytes obtained from patients with progressive multifocal leukoencephalopathy [168–170]. Viral DNA replication was detected in close proximity to PML NBs [169], as were virus particles [171]. Meanwhile, the structural proteins VP1, VP2, and VP3 were reported to accumulate in PML NBs in JCPyV-infected cells [170], and the viral late gene product, the non-structural agnoprotein, participates in this association. In the presence of agnoprotein, the ectopic expression of structural proteins led to the formation of virus-like particles on the surface of PML NBs; however, in its absence, the virus-like particles were predominantly located under the inner nuclear membrane [171]. Thus, it seems that PML NBs provide a scaffold for JCPyV replication and progeny formation. The accumulation of structural proteins inside PML NBs results in the enlargement of the latter [168, 170] and, in the very late phase of infection, their final disruption [168, 169].

Interestingly, an increased number of PML NBs was detected in JCPyV-infected cells early after infection [172]. The authors suggested that in this case, the change in PML NBs number was rather a cellular response to the increased level of interferon induced by the infection than a direct consequence of viral replication.

Altogether, these data suggest that PML NBs may play several roles in PyV infection. Although the absence of PML NBs does not influence viral genome replication, the bodies may still act as scaffolds for virion assembly. Since PML NBs are often re-organized following infection, it is possible that PyVs induce remodeling of the bodies, in order to affect their functionality (e.g., to avoid activation of the innate immune response). Nevertheless, more studies are required to fully understand the complex role of PML NBs and their components in PyV infection.

Box 3. Highlights of the interplay between polyomaviruses and PML NBs

1. The replication of PyV genomes occurs in close proximity to PML NBs.
2. During infection, PML NBs increase in size and number and their composition changes.
3. In MCPyV infection, Sp100 was found to negatively influence viral transcription.
4. In JCPyV infection, particle formation was detected at the surface of PML NBs.

#### Interactions between PML-NB components and hepatitis B virus

Hepadnaviruses are enveloped, small DNA viruses, approximately 42 nm in diameter, with an icosahedral capsid. Compared to all the already mentioned DNA viruses, members of the Hepadnaviridae family are unique in that their DNA genomes are not replicated in the nucleus. Instead, once their genomic DNA has been transcribed in the nucleus, the longest transcript, pregenomic RNA (pgRNA), is encapsidated in the cytoplasm and, at the same time, reverse transcribed into viral DNA, reviewed in [173].

Hepatitis B virus (HBV) has a relaxed circular (rc) dsDNA genome approximately 3.2 kbp in length. Unlike the (−) strand, the (+) strand is not complete. The HBV genome encodes a DNA polymerase (Pol) that has reverse transcriptase activity and is covalently attached to the 5′ end of the (−) strand. The (+) strand has a 5′ short RNA primer. Other proteins encoded by the HBV genome include a capsid protein (also called core protein C or HBcAg), a non-structural secreted.

protein (E; HBeAg), a multifunctional regulatory X protein (HBx), and three surface glycoproteins (HBsAgs) of differing size (small, medium, and large S protein) derived from the same sequence. After transport into the nucleus, HBV rcDNA is converted into covalently closed circular DNA (cccDNA) by host enzymes, and during this process, the above-mentioned 5′ end structure is removed. Transcription of the viral genomic DNA is performed by host RNA Pol II. The template for reverse transcription, pgRNA, also serves for HBcAg and Pol translation. During the early stages of infection, cccDNA can also be amplified in such a way that the newly formed nucleocapsids are, in turn, targeted to the nucleus, where the released genomes are further transcribed, summarized in [46, 173, 174].

Members of the Hepadnaviridae family comprise five genera. They infect mammals (e.g., humans, woodchucks, and ground squirrels), birds (e.g., ducks, geese, and wild herons ), teleost fish, reptiles, and frogs [173, 175]. Human HBV is the prototype and the most-studied member of the Hepadnaviridae family. HBV attacks the liver and can cause both acute and chronic disease, which may result in cirrhosis or even hepatocellular carcinoma (HCC). The World Health Organisation (WHO) estimates that 296 million people were living with chronic hepatitis B infection in 2019, while approximately 830 000 people died, mostly from cirrhosis or HCC [174].

The involvement of PML protein in the early stage of hepatocarcinogenesis and HBV infection and the possible association of HBV infection with PML overexpression or changes in PML NB morphology was revealed by the analysis of human HCC, liver cirrhosis, and chronic hepatitis samples [176]. Meanwhile, Chung and Tsai found an association between HBV genome and PML NBs when studied the progression of HBV-induced HCC [177]. Specifically, the authors reported that chemotherapy and irradiation-induced DNA repair signaling resulted in an increase in PML protein expression as well as the number and size of PML NBs. Moreover, following radiotherapy and chemotherapy, extensive interactions were observed among PML, HDAC1, phosphorylated histone H2AX (γ-H2AX, a marker of dsDNA damage), and breast cancer susceptibility protein type 1 (BRCA1), important for homologous recombination-mediated DNA repair. Additionally, HBcAg and viral DNA were detected in newly formed PML NBs. The authors proposed a model for the feedback loop involving PML, HDAC1, and HBcAg where, in the absence of a stress stimulus, HDAC1 in PML NBs inhibits the basal core promoter of HBV. However, following DNA damage and subsequent repair signaling activation, the expression of pgRNA is enhanced, yielding HBcAg. HBcAg targets PML and disrupts the interaction between HDAC1 and PML, thereby reducing HDAC1 activity and further enhancing viral transcription [177].

##### S100A10 protein recruits HBV polymerase to PML NBs

A yeast two-hybrid screen for cellular protein interacting partners of HBV reverse transcriptase (HBV Pol) led to the identification of S100 calcium-binding protein A10 (S100A10, also known as p11) [45]. The interaction led to the inhibition of DNA Pol activity. Moreover, it was found that S100A10 recruited HBV Pol to the nucleus, where the complex co-localized with PML NBs in an intracellular calcium ion (Ca^2+^)-dependent manner. However, the significance of this interaction is unknown. Whether the S100A10-mediated transport of HBV Pol to PML NBs inhibits viral replication or initiates genome transcription is not clear [45].

##### The FOXO4 transcription factor inhibits transcription from the HBV core promoter

Another relationship between HBV transcription and PML NBs was identified very recently. The transcription of cccDNA from the HBV core promoter can be inhibited by the transcription factor forkhead box O4 (FOXO4) through the FOXO4-mediated downregulation of the expression of another transcription factor, hepatocyte nuclear factor 4 alpha (HNF4α) [178]. The same research group showed that FOXO4 could induce the epigenetic silencing of cccDNA by promoting its heterochromatinization [179]. FOXO4 binds cccDNA and its overexpression decreases the recruitment of euchromatin to cccDNA while increasing that of heterochromatin markers. Interestingly, the authors found that FOXO4 co-localizes with PML NBs and interacts with PML protein. This interaction was shown to be essential for FOXO4-mediated cccDNA epigenetic modification. PML knockdown reversed the FOXO4-mediated epigenetic suppression of cccDNA transcription and, consequently, HBV replication, but did not affect the expression level of HNF4α. These results indicated that the FOXO4-mediated epigenetic suppression of HBV cccDNA transcription and the inhibition of transcription from the core promoter *via* the downregulation of HNF4α are independent processes.

The molecular basis of the role of PML and PML NBs in FOXO4-mediated epigenetic silencing of cccDNA awaits clarification. The same study also revealed that FOXO4 is downregulated in HBV infected primary human hepatocytes and liver biopsy specimens of patients with HBV-positive chronic hepatitis B by an as-yet unidentified mechanism [179].

In the next section, we describe the interactions between the HBV HBx protein and PML NB-associated proteins such as the chromatin remodelers SUZ12 and ZNF198, Sp110, or SMC5/6, which affect the progression of HBV infection.

##### HBx mediates the degradation of SUZ12 and ZNF198, components of PML NBs

Initially, it was shown that HBx mediates the degradation of the PML NB components polycomb protein SUZ12 and zinc finger protein 198 (ZNF198), proteins that have a role in chromatin remodeling. HBx has been found to mediate the activation of the host serine/threonine kinase polo-like kinase 1 (PLK1), which may be associated with cell transformation [180]. SUZ12 and ZNF198 can serve as PLK1 substrates [181]. SUZ12, together with histone-lysine *N*-methyltransferase EZH2 (EZH2) and polycomb protein EED (EED), is a component of polycomb repressive complex 2 (PRC2). The function of this complex is to methylate lysine 27 of histone 3 [H3K27me3], which is associated with the repression of transcription. The ZNF198 protein plays a key role in stabilizing the CoREST complex comprising lysine demethylase 1 (LSD1), REST corepressor 1 (RCOR1), and HDAC1. This large complex is important for its ability to remove activation modifications from chromatin [reviewed in [46]]. The absence of SUZ12 and ZNF198 was observed to lead to defects in DNA repair as well as p53-mediated apoptosis. Interestingly, during HBx-mediated cellular transformation, the level of these proteins decreases, which correlates with an increase in PLK1 levels. Based on these observations, it was proposed that the degradation of SUZ12 and ZNF198 may be related to PLK1 activity [182]. Later, this kinase was shown to phosphorylate SUZ12 and ZNF198, which disrupts their association with the respective complexes, and leads to their ubiquitination-dependent proteasomal degradation [181].

It is important to also mention the long non-coding RNA (lncRNA) HOX antisense intergenic RNA (HOTAIR). Interestingly, this lncRNA functions as a platform for protein ubiquitination through its association with E3 ubiquitin ligases Dzip3 and Mex3b [183]. HOTAIR is thought to facilitate the ubiquitination of phosphorylated SUZ12 and ZFN198 by acting as a bridge between PRC2 and the CoREST complex. The degradation of these complexes may result in the re-expression of some silenced genes, such as epithelial cell adhesion molecule (EpCAM), which may be associated with tumor development. Indeed, the expression of EpCAM is upregulated in some types of HCC [181]. Despite the importance of these findings, further studies are needed to accurately reveal the functional significance of the above-described phenomena.

## HBx mediates the exit of Sp110 protein from PML NBs

Next, it was demonstrated that HBx mediates the exit of Sp110 protein from PML NBs. Sengupta et al. [184] showed that in human hepatoma HepG2 cells infected with HBV, Sp110 becomes deSUMOylated and is released from PML NBs without affecting their integrity. Additionally, the expression of Sp110 is increased. The knockdown of Sp110 resulted in a strong reduction in viral DNA and protein levels and increased the susceptibility of cells to apoptosis, suggesting that Sp110 plays an antiapoptotic role in HBV infection. A comparative microarray analysis of Sp110-depleted *versus* negative control cells revealed that Sp110 regulates (negatively or positively) the expression of many cellular genes involved in immune responses (genes involved in the IFN-response pathway were upregulated), signaling, metabolism, transcription, replication, and DNA repair. A significant number of these genes were found to be direct targets of the HBx protein. Moreover, HBx was found to be required for the exit of Sp110 from PML NBs during HBV infection. Although HBx/PML NB co-localization was not observed, HBx co-localized with redistributed Sp110, as determined by co-immunoprecipitation experiments. The presence of sentrin-specific protease 1 (SENP1), a deSUMOylase, was also detected in the Sp110-HBx complex, and likely mediated the deSUMOylation of Sp110 and, thus, its removal from PML NBs. Sp110 silencing reduces viral replication, apparently by activating the type I IFN-response pathway. The authors concluded that HBx presumably exploits the chromatin-binding properties of Sp110, leading to HBx recruitment to the promoter of cellular genes regulated by Sp110. There, HBx can modulate the recruitment of its associated partners, histone acetylase p300 and HDAC1, thereby positively or negatively affecting the expression of co-regulated cellular genes in a manner favoring viral persistence [184].

## HBx overcomes the SMC5/6-mediated restriction of HBV transcription

The cellular SMC5/6 complex acts as a restriction factor during HBV infection; however, the virus counters this by mediating the degradation of SMC5/6 through HBx, a process that is associated with PML NBs.

The SMC5/6 complex contains six non-SMC subunits (non-structural maintenance of chromosomes element 1 [NSE1], NSE2/MMS21, NSE3, NSE4 (kleisin), NSE5 [SLF1 in humans], and NSE6 [SLF2 in humans]). The NSE1 subunit contains a domain characteristic of ubiquitin E3 ligases and NSE2 is a SUMO E3 ligase. NSE1 and NSE3 contain tandem winged-helix domains and form a subcomplex with the NSE4/kleisin, while NSE5 and NSE6 form heterodimers that bind to either the SMC5/6 nucleotide-binding domain or the hinge domains of Smc5/6 heterodimers [reviewed in [40]]. SMC5/6 is a multifunctional complex with important roles in the maintenance of genome stability (chromosome segregation, replication, and repair), reviewed in [185].

In 2016, two independent studies showed that the SMC5/6 complex functions as a restriction factor of HBV infection [186, 187]. In human hepatocytes, SMC5/6 binds HBV cccDNA and blocks its transcription [186]. Previously, Kanno et al. had demonstrated that SMC5/6 can bind circular DNA as an ATP-dependent DNA linker that functions through the topological entrapment of DNA [188]. HBV counteracts transcription inhibition through the HBx protein. HBx interacts with damage-specific DNA-binding protein 1 (DDB1), a component of the Cullin RING ubiquitin ligase (CRL4) ubiquitin E3 ligase complex. DDB1 binds to exchangeable E3 ligase substrate receptors, DCAFs (DDB1- and CUL4-associated factors) [189]. In HBV-infected cells, HBx binds to DDB1, preventing its binding to cellular DCAFs, and recruits the SMC5/6 complex for ubiquitination and subsequent proteasomal degradation [186, 187]. This mechanism of SMC5/6 complex degradation involving the HBx-DDB1-CUL4 ubiquitin E3 ligase complex was also confirmed in a more recent report [190]; this same study revealed that other cellular HBV restriction factors may also be degraded *via* the same mechanism. Functional testing of HBx proteins from divergent hepadnaviruses that naturally infect primates, rodents, and bats demonstrated that, despite possessing little sequence homology, HBx proteins efficiently degraded mammalian SMC5/6 complexes independently of the host species, and rescued the replication of an HBx-deficient HBV in primary human hepatocytes. These findings indicate that the SMC5/6-mediated defense against hepadnaviruses is evolutionarily conserved [191]. In agreement, the expression of any extrachromosomal plasmid, regardless of the enhancer or promoter sequence, can be suppressed by SMC5/6 and stimulated by HBx expression or SMC5/6 depletion [192].

In non-infected primary human hepatocytes, the SMC5/6 complex is present in PML NBs, co-localizing with two major components of PML NBs, namely, PML and Sp100. The depletion of PML and/or Sp100 leads to the dispersal of the SMC5/6 complex throughout the nucleus and the loss of its restriction activity [193]. Nevertheless, PML and Sp100 are not required for transcription in the presence of HBx, suggesting that these proteins do not affect HBV transcription, but rather function as a scaffold that supports SMC5/6 localization and function.

A very recent study by Abdul et al. shed some light on the molecular steps required for the induction of transcriptional silencing by SMC5/6 and also provided a functional connection between SMC5/6 restriction and PML NBs [194]. The authors reported that both the ATP-binding and ATP-hydrolytic activities of SMC5 and SMC6 are required for episomal silencing. While SMC5/6 binding to chromosomes was reported to involve the interaction of all three components of the NSE1/3/4 paralog a (but not 4b) subcomplex (based on binding activities of their mutant forms), NSE1 and NSE3 mutants retained their episomal silencing ability, which suggests that NSE4a is the only subunit involved in the binding of the SMC5/6 complex to episomal DNA.

These results are in contrast to those of an earlier study by Xu et al., in which the authors demonstrated the involvement of PJA1, a RING-H2 E3 ubiquitin ligase, in facilitating the recognition and binding of the SMC5/6 complex to extrachromosomal DNA. The authors proposed that in the presence of viral and episomal DNA, PJA1 replaces NSE1, yielding a PJA1/NSE3/NSE4 subcomplex that mediated the function of the SMC5/6 complex in the restriction of extrachromosomal DNA transcription [195].

Abdul et al., further revealed that SLF2 (NSE6) is involved in the localization of SMC5/6 to PML NBs, which is essential for SMC5/6-mediated episomal DNA restriction, and also showed that, although NSE2 is not involved in DNA binding, its absence alters the silencing activity of the SMC5/6 complex. In summary, these authors proposed a three-step silencing mechanism whereby (1) the SMC5/6 complex binds to and, likely, topological entraps the episomal DNA template; (2) SMC5/6 (and possibly the associated episomal DNA) is recruited to PML NBs by SLF2; and (3) episomal DNA is silenced *via* a mechanism involving a function of NSE2 other than its SUMO E3 ligase activity [194].

In hepatocytes, HBV is not detected in the early stages of infection and does not induce the innate immune response [193]. However, several host cell proteins that restrict HBV replication have been identified some of which are directly or indirectly related to PML NBs, summarised in [196]. Meanwhile, viral proteins (particularly HBx) interact with components of PML NBs, thereby disrupting or modulating their functions in a manner that favors HBV replication or the induction of HBV persistence and tumorigenic transformation.

Box 4. Highlights of the interplay between hepatitis B virus and PML NBs

1. HBcAg and viral DNA were detected in PML NBs, where HDAC1 inhibits the basal core promoter of HBV. Following the induction of DNA damage responses, the interaction between HDAC1 and PML is disrupted, and viral transcription increases.
2. S100A10 (p11) interacts with HBV Pol and this complex is recruited to PML NBs, leading to the inhibition of DNA Pol activity.
3. FOXO4 interacts with the PML protein, is localized to PML NBs, which leads to the epigenetic suppression of HBV cccDNA.
4. HBx mediates the activation of the serine/threonine kinase PLK1, which promotes the degradation of SUZ12 and ZNF198, components of PML NBs related to chromatin remodelling.
5. HBx protein is required for the exit of Sp110 from PML NBs during HBV infection. HBx is recruited to the promoter of cellular genes regulated by Sp110 likely by exploiting the DNA-binding properties of Sp110.
6. SMC5/6 binds cccDNA, thereby restricting its transcription. For restriction, SMC5/6 is localized to PML NBs. The HBx-DDB1-CUL4 ubiquitin E3 ligase complex targets SMC5/6 for degradation.

### Interactions between PML NB components and anelloviruses

Anelloviridae family members are the most abundant eukaryotic viruses in the human virome. Anelloviruses are vertebrate non-enveloped viruses with icosahedral capsids of approximately 30 nm in diameter. Their genomes are negative-sense, single-stranded, circular DNA approximately 2–4 kb long, that are replicated in the nucleus by a rolling circle mechanism. First, single-stranded DNA is converted into dsDNA by the host DNA Pol, reviewed in [197–199]. The untranslated region of the anellovirus genome contains sequences that may form hairpins, thereby facilitating rolling-circle replication, reviewed in [199]. Metagenomic analyses revealed that anelloviruses are genetically diverse and their virome is present in most human populations, with a prevalence of over 90% [200, 201].They infect humans early in life and replicate persistently, resulting in chronic infections; however, to date, they have not been associated with disease. There is also no evidence of viral clearance following infection. That anellovirus numbers increase during host immunosuppression suggests that they are likely repressed by host immunity [199]. While the adeno-, papilloma-, or polyomaviruses discussed above can induce the S-phase required for the replication of their genomes, anelloviruses lack this ability, and can only replicate in rapidly dividing cells. Torque teno virus (TTV), within the genus *Alphatorquevirus*, was the first identified and is the most studied anellovirus infecting humans [202].

While anelloviral infections in humans are most likely asymptomatic, a member of the genus *Gyrovirus*, chicken anemia virus (CAV), is the causative agent of infectious anemia in young chickens. This disease, which results from the viral-mediated destruction of cortical thymocytes and erythroblasts, is highly lethal to young chickens [203]. The CAV genome produces a single polyadenylated polycistronic transcript with three overlapping open reading frames encoding three viral proteins—VP1, VP2, and VP3 (also called apoptin). VP3 is an inducer of apoptosis. The pro-apoptotic activity of VP3 is cell-specific and is associated with its ability to translocate into the nucleus of transformed cells, but not untransformed ones [204–206]. In chickens carrying tumors induced by Rous sarcoma virus, the intra-tumoral expression of VP3 causes tumor regression [207]. This property makes this protein an attractive target for potential cancer therapy. Poon and co-workers revealed that tumor cell-specific nuclear targeting of VP3 is determined by a bipartite type of nuclear localization signal (NLS 1 and 2), a leucine-rich sequence, a nuclear export signal (NES; amino acids 97–105), and, importantly, a threonine in position 108, adjacent to the NES [208], which is known to be phosphorylated specifically in tumor cells, but not normal ones [209]. The NES is functional in normal but not tumor cells owing to the phosphorylation of threonine 108, which inhibits its action. Poon et al., [208] also demonstrated that a leucine-rich sequence in VP3 (amino acids 33–46) is responsible for its interaction with PML NB components.

VP3 was also shown to interact with the anaphase-promoting complex/cyclosome (APC/C), a major regulator of the cell cycle. The entry of VP3 into the nucleus results in APC/C inhibition, which leads to cell cycle arrest in the G2/M phase, followed by the induction of p53-independent apoptosis [210]. Additionally, in transformed cells, VP3 recruits APC/C to PML NBs. The authors concluded that in transformed cells, cytoplasm/nucleus shuttling is shifted toward nuclear accumulation. VP3 may either transport APC/C subunits from the cytoplasm to the nucleus or associate with APC/C subunits already present in the nucleus and target them to PML NBs. The VP3-mediated recruitment of APC/C to PML NBs in transformed cells leads to the inactivation of APC/C function and the subsequent induction of G2/M-phase arrest and apoptosis [210]. Janssen and co-workers reported that VP3 directly interacts with PML in tumor cells and confirmed that it accumulates in PML NBs. They also showed that VP3 is SUMOylated and that a SUMOylation-deficient VP3 mutant is not recruited to PML NBs, localizing instead to the nuclear matrix. Surprisingly, although the mutant failed to bind PML, it could still induce apoptosis as efficiently as the wild-type protein, and also kill *PML*^−/−^ cells. This observation suggests that VP3 induces apoptosis in transformed cells independently of PML and SUMOylation [211].

In summary, the role of PML NBs in VP3-induced apoptosis, as well as the significance of the interaction between VP3 and PML and VP3 SUMOylation remain unknown and merit further investigation.

Box 5. Highlights of the interplay between anelloviruses and PML NBs

1. VP3 interacts with the anaphase-promoting complex/cyclosome (APC/C), a major regulator of the cell cycle, and recruits it to PML NBs in transformed cells. This leads to the inactivation of APC/C function and the subsequent induction of G2/M-phase arrest and apoptosis.
2. VP3 interacts with PML protein and is SUMOylated.
3. The role of PML NBs in VP3-induced apoptosis, as well as the significance of VP3 SUMOylation, remain unclear.

## Conclusions

PML NBs participate in numerous cellular processes. Although they are dispensable for cell survival, their importance is highlighted by various cellular disorders associated with their dysregulation. Our understanding of the function of PML NBs has evolved considerably since their discovery 60 years ago. Nevertheless, many molecular details relating to the functions of PML NBs are still elusive, in great part owing to their extremely complex and dynamic composition. Moreover, the composition of PML NBs is tissue-specific, which adds further complexity to their investigation. Research on the interplay between individual PML NB components and viruses contributes not only to our understanding of the role of PML NBs during infection but also to the elucidation of their physiologic functions in cells.

**Fig. 2:**
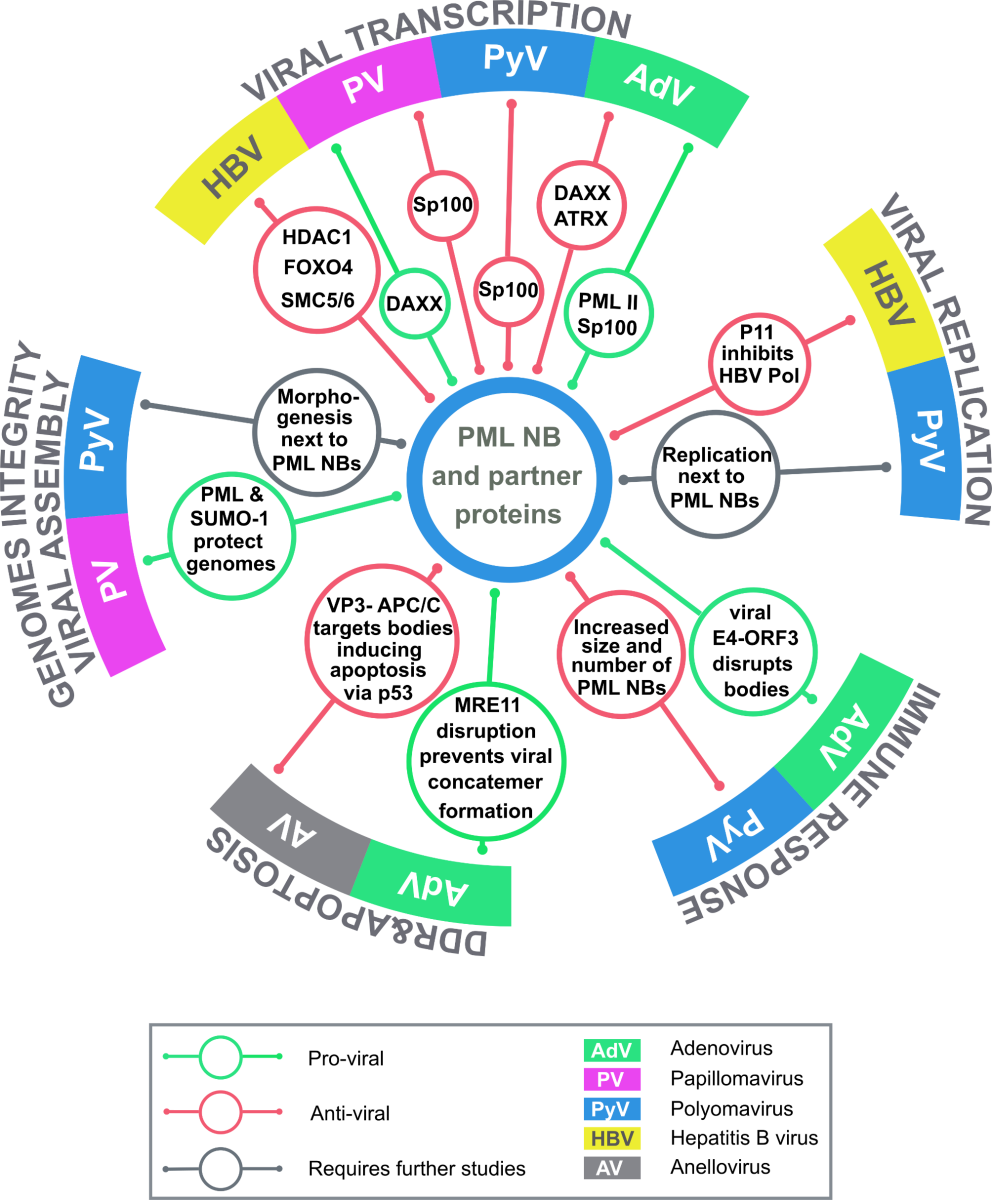
PML NBs and their components that interact with small and medium size DNA viruses - an overview

From the review, we can conclude that the main interactions between PML NBs and small and medium size DNA viruses affect viral genome transcription, integrity, replication and virion assembly or, influence cell responses such as DNA damage, apoptosis and innate immune response activation. The main interactions between PML NBs or their components and the viruses discussed in the review are presented in the Fig. 2.

The role of PML NBs in the life cycle of small and medium size DNA viruses has been most extensively studied in PVs and AdVs. Intriguingly, while PVs were found to utilize PML NBs mainly to their advantage, the opposite has been described for AdVs. This may be partly due to the different infectious strategies used by the viruses, namely, lytic (AdVs) and persistent (PVs). Meanwhile, several aspects of the functional significance of interactions between PML NB components and HBV proteins require further confirmation, and the consequences of the positioning of polyomavirus genomes, which are transcribed and replicated in the immediate vicinity of PML NBs, need to be explored in detail. Accordingly, in future studies, it is particularly important to identify the mechanisms by which PML NB-associated chaperones and remodeling proteins affect the composition of viral minichromosomes and regulate transcription either soon after the viral genomes appear in the nucleus, or late in the infection when transcription and replication must be stopped to allow virion assembly. Furthermore, as the DNA damage response is tightly coupled to PML NBs and small DNA viruses (e.g., polyomaviruses) activate DDR mechanisms, the relationship between DDR and PML NBs and its impact on virus propagation or establishment of latency also requires further investigation.

A major challenge in understanding the biological function of PML bodies is their dynamic composition and movement. For example, recent studies have shown that PML NB fission in response to DNA [81] requires the polymerization of nuclear actin for both PML NB fission and the repositioning of the NBs bodies at sites of DNA lesions [212]. Therefore, to understand the diverse roles of the PML NB components relating to viral entry into the nucleus, transcription, replication, genome repair, and assembly, it is essential that fluorescence live-cell imaging in combination with super-resolution techniques is employed in future studies. Such studies will be even more beneficial if accompanied by a biological analysis of interactions between PML NBs and viral components and their post-translation modifications, such as the SUMOylation of PML NBs and/or interacting viral components.

Finally, most studies investigating interactions between PML NBs and viruses were undertaken without considering the influence of innate immunity-related factors. Given that PML NBs are both interferon-regulated and interferon-regulating structures, it will be interesting to study changes in the composition of PML NBs while controlling (restricting or activating) IFN responses at different time points of the viral life cycle.

## Data Availability

Not applicable.

## List of Abbreviations

3D: Three-dimensional
3T6: Mouse fibroblast cell line
AdV: Adenovirus
APC/C: Anaphase-promoting complex/cyclosome
ATP: Adenosine triphosphate
ATRX: α-thalassemia mental retardation syndrome X-linked protein
BKPyV: BK polyomavirus
BPV: Bovine papillomavirus
BRCA1: Breast cancer type 1 susceptibility protein
CAV: Chicken anemia virus
cccDNA: Covalently closed circular DNA
CoREST: REST corepressor 1
cGAS: Cyclic GMP–AMP synthase
CRL4: Cullin RING ubiquitin ligase
CRL4WDR4: WD repeat 4–containing cullin-RING ubiquitin ligase 4
Cul: Cullin
DAPI: 4’,6-diamidin-2-fenylindol
Daxx: Death-domain associated protein
DCAF: DDB1- and CUL4-associated factor
DDB1: Damage-specific DNA-binding protein 1
DDR: DNA damage response
DNA: Deoxyribonucleic acid
dsDNA: Double-stranded DNA
Dzip3: DAZ interacting Zinc finger protein 3
E: Early
E6AP: E6-associated protein, ubiquitin ligase
EdU: 5-Ethynyl-2′-deoxyuridine
EED: Polycomb protein EED
EGFP: Enhanced green fluorescent protein
EpCAM: Epithelial cell adhesion molecule
EZH2: Histone methyltransferase EZH2
FOXO4: Transcription factor forkhead box O4
GFP: Green fluorescent protein
H: Histone
HaCaT: Immortalized human keratinocytes
H2AX: H2A histone family member X
HATs: Histone acetyltransferases
HBcAg: Hepatitis B core antigen
HBeAg: Hepatitis B envelope antigen
HBsAg: Hepatitis B surface antigen
HBV: Hepatitis B virus
HBx: Hepatitis B virus X protein
HCC: Hepatocellular carcinoma
HDAC: Histone deacetylase
HDAC1: Histone deacetylase 1
HNF4α: Hepatocyte nuclear factor 4 alpha
HOTAIR: HOX transcript antisense intergenic RNA
HP1: Heterochromatin protein 1
HP1α: Heterochromatin protein 1 alpha
HPV: Human papillomavirus
HSV-1: Herpes simplex virus 1
I: Intermediate
ICP0: Regulatory protein HSC-1
IFN: α/β/γ Interferon α/β/γ
JCPyV: JC polyomavirus
kbp: Kilobase pairs
L: Late
LINE-1: Long interspersed nuclear element-1
lncRNA: Long non-coding RNA
LSD1: Lysine demethylase 1
LT: Large T antigen
Lys: Lysine
MCPyV: Merkel cell polyomavirus
Mex3B: Mex-3 RNA binding family member B
MPyV: Murine polyomavirus
Mre11: Meiotic recombination 11 protein
MRN: Complex Mre11-Rad50-Nbs1
NB: Nuclear body
Nbs1: Nijmegen breakage syndrome 1 protein, Nibrin
NES: Nuclear export signal
NLS: Nuclear localization sequence
NSE: Non-structural maintenance of chromosomes element
ORF: Open reading frame
p53: p53 protein
p62: p62 protein
p73: p73 protein
PJA1: Praja ring finger ubiquitin ligase 1
pgRNA: Pregenomic RNA
PIAS1: Protein inhibitor of activated STAT 1
PIAS3: Protein inhibitor of activated STAT 3
Plk1: Polo like kinase
PML: Promyelocytic leukemia
PML: NBs Promyelocytic leukemia nuclear bodies
Pol: Polymerase
PRC2: Polycomb repressive complex 2
PV: Papillomavirus
PyV: Polyomavirus
Rad50: DNA repair protein Rad50
RanBP2: RAN binding protein 2
RBCC: RING-B box-Coiled Coil
Rbx1: Ring-box 1 protein
rc: Relaxed circular
rcDNA: Relaxed circular double-stranded DNA
RCOR1: REST corepressor 1
RCs: Replication centers
REST: RE1-silencing transcription factor
RING: Really interesting new gene
RNA: Ribonucleic acid
RNA pol II: RNA polymerase II
RNF4: RING finger protein 4
ROS: Reactive oxygen species
S100A10: S100 calcium binding protein A10
SENP1: Sentrin -specific protease 1
SLF2: SMC5-SMC6 Complex Localization Factor 2
SIAH1: Seven in absentia homolog 1 protein
SIAH2: Seven in absentia homolog 2 protein
SIM: SUMO-binding motif
Smc5/6: Structural maintenance of chromosomes protein 5/6
Sp100: Speckled protein 100 kilodaltons
Sp100A: Speckled protein 100 kilodaltons isoform A
Sp100B: Speckled protein 100 kilodaltons isoform B
Sp100C: Speckled protein 100 kilodaltons isoform C
Sp100-HMG: Speckled protein 100 kilodaltons isoform HMG
Sp110: Speckled protein 110 kilodaltons
S-phase: Synthesis phase
STED: Stimulated emission depletion
STING: Stimulator of interferon genes
SUMO: Small ubiquitin-like modifier
SUZ12: Polycomb repressive complex 2 subunit
TGN: Trans-Golgi network
TRIM: Tripartite motif
TTV: Torque teno virus
U2OS: Human osteosarcoma cell line
Ubc9: Ubiquitin-conjugating enzyme 9
UHRF1: Ubiquitin Like With PHD And Ring Finger Domains 1
vDNA: Viral DNA
VP1/2/3: Viral protein 1/2/3
WD: Tryptophan-aspartic acid (W-D) dipeptide motif
WHO: World Health Organization
ZNF198: Zinc finger protein 198
ZNF451: Zinc finger protein 451
YAP: Yes-associated protein
γH2AX: Phosphorylated H2AX
